# Enhancing the Photocatalytic Activity of Lead-Free Halide Perovskite Cs_3_Bi_2_I_9_ by Compositing with Ti_3_C_2_ MXene

**DOI:** 10.3390/molecules29215096

**Published:** 2024-10-28

**Authors:** Tao Tang, Xiaoyu Dou, Haoran Zhang, Hexu Wang, Ming Li, Guanghui Hu, Jianfeng Wen, Li Jiang

**Affiliations:** 1 Key Laboratory of Low-Dimensional Structural Physics and Application, Education Department of Guangxi Zhuang Autonomous Region, College of Physics and Electronic Information Engineering, Guilin University of Technology, Guilin 541004, China; tangtao@glut.edu.cn (T.T.); douxiaoyu1018@163.com (X.D.); glzhanghaoran@163.com (H.Z.); wanghx0907@163.com (H.W.); liming928@163.com (M.L.); guanghui@glut.edu.cn (G.H.); 2 School of Electronic Information and Automation, Guilin University of Aerospace Technology, Guilin 541004, China

**Keywords:** photocatalysis, lead-free perovskite, Cs_3_Bi_2_I_9_, Ti_3_C_2_

## Abstract

In recent years, halide perovskite materials have become widely used in solar cells, photovoltaics, and LEDs, as well as photocatalysis. Lead-free perovskite Cs_3_Bi_2_I_9_ has been demonstrated as an effective photocatalyst; however, the fast recombination of the photogenerated carriers hinders further improvements of its photocatalytic activity. In this work, Ti_3_C_2_ was composited with Cs_3_Bi_2_I_9_ to promote the transfer and separation of photogenerated carriers, and thus the pollutant degradation efficiency was effectively improved. The visible-light photocatalytic reduction of Cs_3_Bi_2_I_9_/Ti_3_C_2_ on rhodamine B (RhB), methylene blue (MB), and malachite green (MG) was as high as 97.3%, 96%, and 98.8%, respectively, improvements of almost 31.2%, 37.8%, and 37.2% compared to that of sole Cs_3_Bi_2_I_9_. Our study provides a simple way to enhance the photocatalytic activity of lead-free halide perovskites.

## 1. Introduction

In recent years, carcinogenic organic pollutants have been directly discharged into industrial wastewater, especially rhodamine B (RhB), methylene blue (MB), and malachite green (MG), so the issue of how to remove these pollutants via an effective and economic method has become one of the most popular topics [1,2,3,4,5]. Currently, common methods for treating organic pollutants include adsorption, electrocatalytic degradation, ultrasonic method, ultraviolet (UV) irradiation, and non-homogeneous Fenton-like oxidation. Without shortcomings such as high cost, secondary pollution, and long process flow, photocatalytic degradation of organic pollutants is one of the most efficient, practical, and environmentally friendly methods [6,7].

In the process of organic pollutants photodegradation, the semiconductor photocatalyst acts as the core to absorb light of a specific wavelength, which makes the electrons of the photocatalyst leap from the valence band (VB) to the conduction band (CB), and generate holes in the VB. The electrons in the CB combine with oxygen to form the superoxide radicals (•O_2_^−^), and the holes in the VB combine with the water molecules to form the hydroxyl radicals (•OH). Eventually, organic pollutants react with •O_2_^−^ and •OH radicals to decompose into mineralized compounds. Many semiconductor materials, typically including TiO_2_ [8], ZnO [9], g-C_3_N_4_ [10], and CsPbX_3_ [11], have been used as popular photocatalysts; however, due to the wide band gap of TiO_2_, ZnO and g-C_3_N_4_, they can only absorb UV or only a small part of visible light and have low photocatalytic activity. Recently, CsPbX_3_ has been demonstrated as an efficient visible-light photocatalyst, but the toxicity of lead and its instability in water limit its photocatalytic applications [12]. Although the water stability can be improved by surface functionalization [11] and water-resistant coatings [13], developing a water-stable lead-free halide perovskite photocatalyst is more important. Recently, non- or low-toxicity Sn- and Bi-based materials have been widely realized in the halide perovskite family [14,15,16,17,18]. However, compared to Pb-based perovskites, Sn-based halide perovskites are inherently unstable and have low photocatalytic efficiency [19]. In contrast, Bi^3+^ has a similar electronic configuration and ionic radius to Pb^2+^, and Bi-based halide perovskites have a higher water stability than Pb-based ones, so currently they have become the star materials for replacing Pb-based perovskites in photocatalysis [20,21], let alone their lesser toxicity due to their stable +3 valence oxidation state [22]. Moreover, Bi-based halide perovskites, especially Cs_3_Bi_2_X_9_ (X = Cl, Br, I), have been widely used in solar cells [23,24], light-emitting diodes [25], bio-imaging [26], and other fields.

The band gap of ~2.35 eV of Cs_3_Bi_2_I_9_ makes it attractive in the field of visible-light photocatalysis [27,28,29]. However, the high exciton binding energy of Cs_3_Bi_2_I_9_ hinders the effective separation of photoelectrons and holes [30,31]. Encapsulating Cs_3_Bi_2_I_9_ with CeO_2_ [15] or Bi_2_WO_6_ [16] can facilitate the separation and transfer of photocarriers; nevertheless, the photocatalytic performance of these Bi-based halide perovskites is still unsatisfactory, possibly due to the small specific surface area of these materials. Therefore, it is worthwhile to find a material with large specific surface area, which is helpful to rapidly separate and transfer the photo-excited electrons and holes in Cs_3_Bi_2_I_9_, so as to improve the photocatalytic performance. In recent years, Ti_3_C_2_ MXene has been widely used as adjuvant in pollutant photodegradation due to its simple preparation, low cost, good stability, large specific surface area, excellent electrical conductivity, high carrier mobility, and abundant active centers [32,33,34]. When composited with semiconductor photocatalysts, Ti_3_C_2_ supplies abundant charge transfer channels due to the large contact area, which is conducive to the effective transfer and separation of photogenerated carriers [35,36,37,38,39] and thus greatly improves the photocatalytic efficiency.

In this work, Cs_3_Bi_2_I_9_/Ti_3_C_2_ photocatalysts were synthesized by the thermal injection method, and their aqueous photocatalytic degradation performances on RhB, MB, and MG were investigated. Cs_3_Bi_2_I_9_/Ti_3_C_2_ photocatalyst could be perfectly dispersed in water for 30 days without any change of its water stability. Under visible light, Cs_3_Bi_2_I_9_ can photodegrade 97.3% RhB (20 mg/L), 96.0% MB (10 mg/L), and 98.8% MG (20 mg/L) in 30 min, respectively. After three cycle experiments, the photocatalytic activity decreases very little. The fast transfer of photoelectrons from Cs_3_Bi_2_I_9_ to Ti_3_C_2_ is deemed accountable for the photocatalytic improvement. We highlight the importance and effectiveness of the combination of non-lead halide perovskites and MXenes in pollutant photodegradation.

## 2. Results

**Figure 1:**
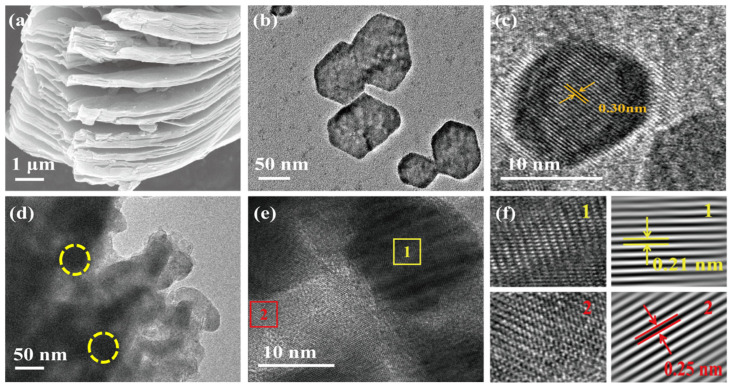
(**a**) SEM image of Ti_3_C_2_. (**b**) TEM and (**c**) high-resolution TEM images of Cs_3_Bi_2_I_9_. (**d**) TEM and (**e**) high-resolution TEM images of Cs_3_Bi_2_I_9_/Ti_3_C_2_ composite. (**f**) Lattice streak diagram of Cs_3_Bi_2_I_9_/Ti_3_C_2_ composite.

The morphology of the accordion-multilayered Ti_3_C_2_ material can be observed in Figure 1a with a uniform thickness [40]. In Figure 1b, a TEM image of a separate Cs_3_Bi_2_I_9_ crystal is exhibited as a hexagonal structure with a size of about 75 nm [41]. Figure 1c shows a high-resolution TEM image of Cs_3_Bi_2_I_9_, with the 0.30 nm spacing corresponding to the (204) crystal planes. Figure 1d shows a TEM image of the Cs_3_Bi_2_I_9_/Ti_3_C_2_ composite with Cs_3_Bi_2_I_9_ embedded on the surface of Ti_3_C_2_. The different crystal structures of Cs_3_Bi_2_I_9_ and Ti_3_C_2_ can be clearly resolved in Figure 1e,f (see square 1 and 2 in the insets). The 0.21 and 0.25 nm spacings should be ascribed to the (220) crystal planes of Cs_3_Bi_2_I_9_ and the (011) crystal planes of Ti_3_C_2_, respectively. The above results indicate that the Cs_3_Bi_2_I_9_ nanocrystals combine well with the Ti_3_C_2_ material.

**Figure 2:**
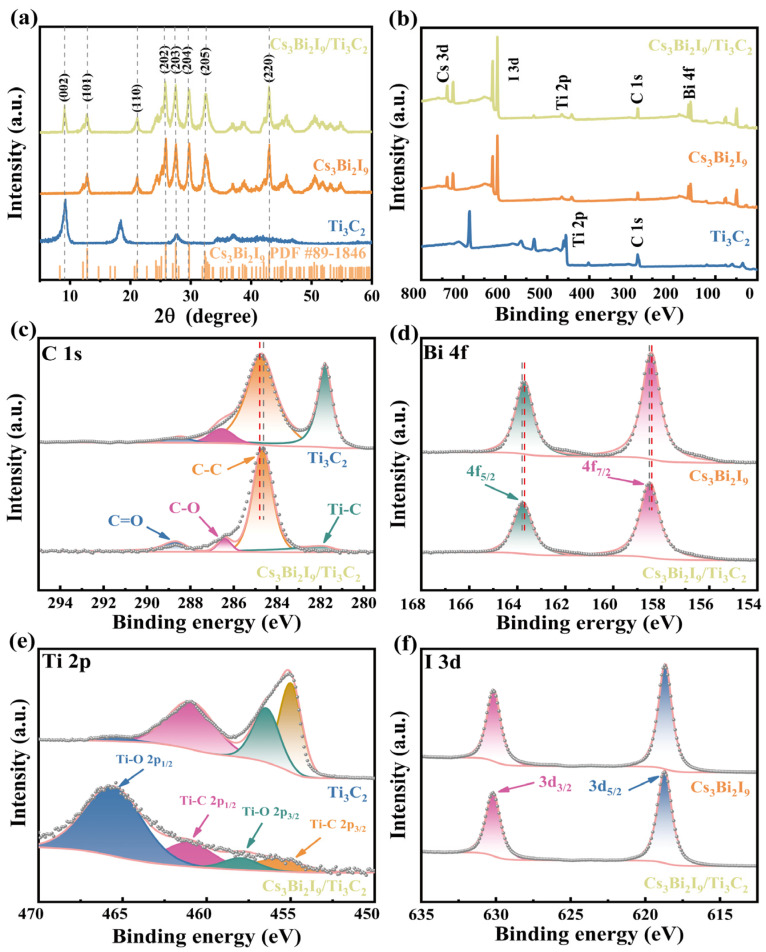
(**a**) XRD and (**b**) XPS spectra of Ti_3_C_2_, Cs_3_Bi_2_I_9_, and Cs_3_Bi_2_I_9_/Ti_3_C_2_. (**c**) C 1s and (**e**) Ti 2p spectra of Ti_3_C_2_ and Cs_3_Bi_2_I_9_/Ti_3_C_2_. (**d**) Bi 4f and (**f**) I 3d spectra of Cs_3_Bi_2_I_9_ and Cs_3_Bi_2_I_9_/Ti_3_C_2_ composites, respectively.

**Figure 3:**
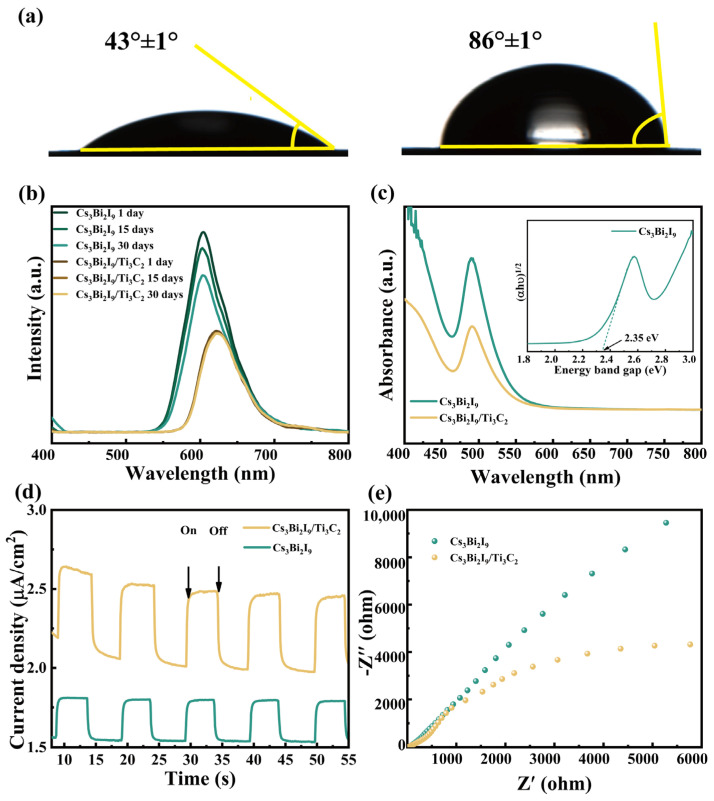
(**a**) Water contact angles of Cs_3_Bi_2_I_9_ and Cs_3_Bi_2_I_9_/Ti_3_C_2_. (**b**) Time-dependent PL spectra of Cs_3_Bi_2_I_9_ and Cs_3_Bi_2_I_9_/Ti_3_C_2_ aqueous solutions. The excitation wavelength is 390 nm. (**c**) UV-vis absorption spectra of Cs_3_Bi_2_I_9_ and Cs_3_Bi_2_I_9_/Ti_3_C_2_. The inset is the Tauc plots of Cs_3_Bi_2_I_9_. (**d**) Photocurrent responses and (**e**) electrochemical impedances of Cs_3_Bi_2_I_9_ and Cs_3_Bi_2_I_9_/Ti_3_C_2_.

The crystal structures of Cs_3_Bi_2_I_9_, Ti_3_C_2_, and Cs_3_Bi_2_I_9_/Ti_3_C_2_ composites were analyzed using XRD. In Figure 2a, it can be seen that the XRD pattern of Cs_3_Bi_2_I_9_ is fully consistent with the standard PDF#89-1846 of Cs_3_Bi_2_I_9_ [42], and the XRD pattern of Ti_3_C_2_ is in agreement with what has been reported in the previous literature [43]. In addition, the characteristic diffraction peaks at 12.84°, 21.11°, 25.84°, 27.52°, 29.72°, 32.35°, and 42.98° correspond to the (101), (110), (203), (204), (205), and (220) crystal planes of Cs_3_Bi_2_I_9_, respectively, and the typical diffraction peak present at 8.71° represents the (002) crystal planes of Ti_3_C_2_. No new peaks were observed, which indicates that Cs_3_Bi_2_I_9_ and Ti_3_C_2_ were merely physically mixed. XPS analyses were used to characterize the elemental composition and valence states of the samples. As shown in Figure 2b, typical peaks of Ti 2p and C 1s were observed in the Cs_3_Bi_2_I_9_/Ti_3_C_2_ composites, which belong to Ti_3_C_2_ [44]. Typical peaks of Cs 3d, Bi 4f, and I 3d were detected, which belong to Cs_3_Bi_2_I_9_. In addition, the carbides inevitably contain oxygen since they are generally synthesized in air [40]. According to the high-resolution C 1s XPS spectra (Figure 2c), four subpeaks can be seen, which are C=O, C–O, C–C, and Ti–C. As shown in Figure 3e, the high-resolution Ti 2p XPS spectra can be deconvoluted into four subpeaks, namely Ti–O 2p_1/2_, Ti–C 2p_1/2_, Ti–O 2p_3/2_, and Ti–C 2p_3/2_ [45]. The intensity variation of the subpeaks of C 1s and Ti 2p in Cs_3_Bi_2_I_9_/Ti_3_C_2_ should be ascribed to the reheating of Ti_3_C_2_ during the synthesis process (see Section 4.3). It is noteworthy that compared to sole Ti_3_C_2_ and Cs_3_Bi_2_I_9_, in Cs_3_Bi_2_I_9_/Ti_3_C_2_, the binding energies of C 1s (Figure 2c), Bi 4f (Figure 2d), and Ti 2p (Figure 2e) were slightly shifted, and I 3d (Figure 2f) remained static. This may be due to the carrier transfer between Cs_3_Bi_2_I_9_ and Ti_3_C_2_ through its heterogeneous interface [46].

The water stability of the photocatalyst is a key factor determining whether photocatalysis can be performed in water. It can be observed from Figure 3a that the contact angles of water on Cs_3_Bi_2_I_9_ and Cs_3_Bi_2_I_9_/Ti_3_C_2_ are 43° ± 1° and 86° ± 1°, respectively. A larger contact angle of Cs_3_Bi_2_I_9_/Ti_3_C_2_ usually means it has a smaller contact area with water molecules. Although the lower hydrophilicity of Cs_3_Bi_2_I_9_/Ti_3_C_2_ cannot directly determine its better water stability, to a certain extent it also reduces the possibility of water erosion. Additionally, as shown in Figure 3b, at an excitation wavelength of 390 nm, Cs_3_Bi_2_I_9_/Ti_3_C_2_ has a broad emission peak at 540–700 nm, and the shape and intensity of the emission peaks are almost unchanged, which demonstrates that Cs_3_Bi_2_I_9_/Ti_3_C_2_ can be stably dispersed in water for at least 30 days. On the other hand, the emission intensity of Cs_3_Bi_2_I_9_ was obviously weakened with time, indicating that the stability of Cs_3_Bi_2_I_9_/Ti_3_C_2_ in water is higher than that of Cs_3_Bi_2_I_9_. The emissive center of pure Cs_3_Bi_2_I_9_ is located at 602 nm, and when Ti_3_C_2_ and Cs_3_Bi_2_I_9_ form a heterostructure, it redshifts to 622 nm. The emissive intensity is also weaker after the combination of Ti_3_C_2_ and Cs_3_Bi_2_I_9_, implying a quantitative decrease in carriers when undergoing radiative recombination.

The UV-vis absorption spectra of Cs_3_Bi_2_I_9_ and Cs_3_Bi_2_I_9_/Ti_3_C_2_ and the corresponding Tauc plots are shown in Figure 3c. They both exhibit distinct absorption peak at ~ 493 nm and a long absorption tail extending to ~600 nm, indicating the strong visible-light capture ability of Cs_3_Bi_2_I_9_. The band gap of Cs_3_Bi_2_I_9_ is assumed to be ~2.35 eV (see the inset of Figure 3c). The photocurrent results are shown in Figure 3d. Under the same illumination, the photocurrent of Cs_3_Bi_2_I_9_/Ti_3_C_2_ is obviously larger than Cs_3_Bi_2_I_9_, which demonstrates that more free carriers were generated due to a better separation of photogenerated e-h pairs as well as a faster carrier transfer ability of Cs_3_Bi_2_I_9_/Ti_3_C_2_. In Figure 3e, Cs_3_Bi_2_I_9_/Ti_3_C_2_ has a smaller electrochemical impedance than Cs_3_Bi_2_I_9_, again showing the higher charge transfer rate of Cs_3_Bi_2_I_9_/Ti_3_C_2_. Therefore, based on the optical and photoelectrical measurements, it is known that after the composition of Ti_3_C_2_, Cs_3_Bi_2_I_9_ shows a distinct improvement of the carrier separation and transfer, which is beneficial to photocatalysis.

**Figure 4:**
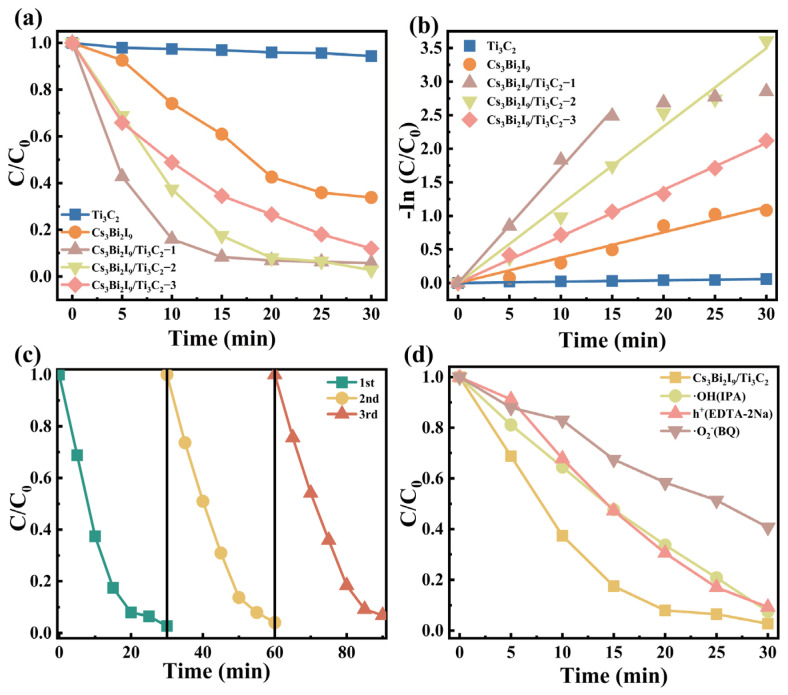
(**a**) Effects of Cs_3_Bi_2_I_9_, Ti_3_C_2_, Cs_3_Bi_2_I_9_/Ti_3_C_2_-1, Cs_3_Bi_2_I_9_/Ti_3_C_2_-2, and Cs_3_Bi_2_I_9_/Ti_3_C_2_-3 on the photocatalytic degradation of RhB under visible light. (**b**) The corresponding degradation kinetic behavior. (**c**) Photocatalytic cycle tests of Cs_3_Bi_2_I_9_/Ti_3_C_2_. (**d**) Effects of scavengers on the photocatalytic activity of Cs_3_Bi_2_I_9_/Ti_3_C_2_.

The photocatalytic degradation of organic pollutants was carried out with Ti_3_C_2_, Cs_3_Bi_2_I_9_, and Cs_3_Bi_2_I_9_/Ti_3_C_2_. Samples with different mass ratios of Ti_3_C_2_ were also used for comparison. We tuned the amount of Ti_3_C_2_ in the Cs_3_Bi_2_I_9_/Ti_3_C_2_ synthesis process (see Section 4.3) with 3, 5, and 7 mg, and named them as Cs_3_Bi_2_I_9_/Ti_3_C_2_-1, Cs_3_Bi_2_I_9_/Ti_3_C_2_-2, and Cs_3_Bi_2_I_9_/Ti_3_C_2_-3, respectively. All the photocatalysts were first stirred in dark for 30 min to reach the adsorption–desorption equilibrium. The photodegradation ability is evaluated by the ratio of the real-time and initial concentrations (C/C_0_) of organic dyes. As shown in Figure 4a, after 30 min of light exposure, Ti_3_C_2_, Cs_3_Bi_2_I_9_, Cs_3_Bi_2_I_9_/Ti_3_C_2_-1, Cs_3_Bi_2_I_9_/Ti_3_C_2_-2, and Cs_3_Bi_2_I_9_/Ti_3_C_2_-3 could degrade 6%, 66.1%, 94.0%, 97.3%, and 88.0% of RhB, respectively. The photocatalytic activity of the sole Ti_3_C_2_ can almost be neglected, and the composite of Cs_3_Bi_2_I_9_/Ti_3_C_2_ is greatly superior to the sole Cs_3_Bi_2_I_9_. The photocatalytic ability decreases instead when the mass of Ti_3_C_2_ increased from 5 to 7 mg, probably because too much Ti_3_C_2_ blocks the effective light absorption of Cs_3_Bi_2_I_9_. The corresponding photodegradation kinetics were fitted using the first-order reaction equation:

$$-\ln(\frac{C}{C_{0}})=\mathrm{kt}$$

where k is the photocatalytic efficiency (in this discussion its unit min^−1^ is omitted), and as long as the photodegradation limit is achieved, the subsequent data are not used for linear fitting. As shown in Figure 4b, the k value of Cs_3_Bi_2_I_9_/Ti_3_C_2_-2 is 0.12, which is 3.08 times higher than that of Cs_3_Bi_2_I_9_. It is worth noting that the k value of Cs_3_Bi_2_I_9_/Ti_3_C_2_-1 is the largest (0.17), but in a mere 15 min, it quickly approaches the photodegradation limit, so the final dye depollution ratio is less than that of Cs_3_Bi_2_I_9_/Ti_3_C_2_-2 (Figure 4a).

**Figure 5:**
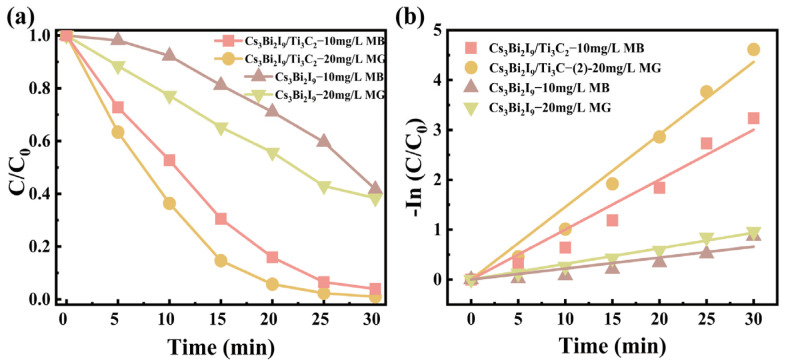
(**a**) Effects of Cs_3_Bi_2_I_9_ and Cs_3_Bi_2_I_9_/Ti_3_C_2_ on photocatalytic degradation of MB and MG under visible light. (**b**) Corresponding degradation kinetic behaviors.

In order to further evaluate the catalytic practicality of the composites, three cycle experiments were conducted. As shown in Figure 4c, the photocatalytic ability of Cs_3_Bi_2_I_9_/Ti_3_C_2_ is very stable, and only a very small drop due to the recycling mass loss can be found. To make clear the mechanism of the photocatalytic reaction, free radical trapping agent experiments were performed. As shown in Figure 4d, isopropyl alcohol (IPA), ethylenediamine tetraacetic acid disodium salt (EDTA-2Na), and benzoquinone (BQ) were used to scavenge •OH, h^+^, and •O_2_^−^, respectively [47]. The photocatalytic activity of the composites showed a small decrease when IPA and EDTA-2Na were added, while it decreased significantly when BQ was used, which indicated that •O_2_^−^ radicals were mainly involved in the photocatalytic reaction. Regrettably, several capture agents (including AgNO_3_, K_2_Cr_2_O_7_, and KBrO_3_) were used to capture electrons, and it was found that RhB was rapidly adsorbed, making it hard to assess the photodegradation ability after electron capture. In addition, Cs_3_Bi_2_I_9_/Ti_3_C_2_-2 can also effectively photodegrade other organic pollutants. As shown in Figure 5a, it can degrade 96.0% of MB and 98.8% of MG in 30 min, obviously better than sole Cs_3_Bi_2_I_9_ (58.2% on MB and 61.9% on MG). Furthermore, the photodegradation kinetics were fitted to the time (Figure 5b), and the obtained k values are 0.10 and 0.15, respectively, roughly 4.56 and 4.65 times those of Cs_3_Bi_2_I_9_.

In addition, Table 1 compares the photocatalytic degradation of organic pollutants of Cs_3_Bi_2_I_9_/Ti_3_C_2_ with other halide perovskites. Many halide perovskites can effectively degrade organic pollutants, which indicates that halide perovskites have a good future in the photocatalytic field. However, due to the ubiquitous aqueous instability of Pb- and Sn-based perovskites, photocatalytic degradation of pollutants in previous studies is generally carried out in organic solution, and only a small part is carried out in water after water-resistance functionalization [48,49,50,51]. It can be seen in Table 1 that Cs_3_Bi_2_X_9_ can achieve a better photocatalytic effect than lead perovskites, especially for high-concentration organic pollutants. Moreover, its water stability is good enough, and it could even be directly used in water [14,52,53,54]. After composition with g-C_3_N_4_ or Ti_3_C_2_, the visible-light photocatalytic activity of Cs_3_Bi_2_I_9_ is obviously improved, and Cs_3_Bi_2_I_9_/Ti_3_C_2_ is significantly better than Cs_3_Bi_2_I_9_/g-C_3_N_4_. Since g-C_3_N_4_ is a luminescent semiconductor and Ti_3_C_2_ is almost inert to light, it is reasonably to conclude that the radiative recombination of e-h pairs in g-C_3_N_4_ is accountable for the relatively poor effect of Cs_3_Bi_2_I_9_/g-C_3_N_4_.

## 3. Discussion

**Figure 6:**
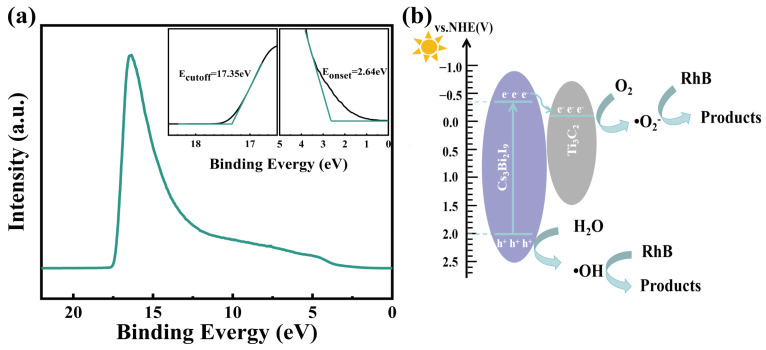
(**a**) The UPS measurements of Cs_3_Bi_2_I_9_, and inset are the onset and cutoff energies. (**b**) Schematic of photocatalytic mechanism of Cs_3_Bi_2_I_9_/Ti_3_C_2_ under visible light.

A schematic diagram of the photocatalytic mechanism of Cs_3_Bi_2_I_9_/Ti_3_C_2_ under visible light is shown in Figure 6. In Figure 6a, taking the vacuum as a reference, the VB Maximum (E_VBM_) can be calculated by conducting the UPS measurements, i.e., E_VBM_ = −[21.22 − (E_cutoff_ − E_onset_)]; here, 21.22 eV is the photon energy of He I emission of the UPS equipment. For Cs_3_Bi_2_I_9_, E_cutoff_ is 17.35 eV, and E_onset_ is 2.64 eV, so that E_VBM_ is found to be −6.51 eV. Considering that the bandgap is 2.35 eV, we thus obtain the CB minimum (E_CBM_) as −4.16 eV. Generally, we use the normal hydrogen electrode (NHE) as the reference electrode to represent the energy level, i.e., E(NHE) = −4.5 − E(vacuum), to represent the relationship between NHE and vacuum energy level, thus obtaining the values of VBM and CBM as 2.01 eV and −0.34 eV, respectively. In addition, the Fermi energy level of the Ti_3_C_2_ material is located at −0.13 eV [56], so the photogenerated electrons in Cs_3_Bi_2_I_9_ can be spontaneously transferred to Ti_3_C_2_ [Figure 6b]. Therein, a rational photocatalytic process was proposed to illustrate how the Cs_3_Bi_2_I_9_/Ti_3_C_2_ photocatalyst photodegrades RhB pollutant:

(1) $${\mathrm{Cs}}_{3}{\mathrm{Bi}}_{2}I_{9}+h\nu \;\to \;e^{-}\;+\;h^{+}$$

(2) $${\mathrm{Cs}}_{3}{\mathrm{Bi}}_{2}I_{9}(e^{-})+{\mathrm{Ti}}_{3}C_{2}\;\to \;{\mathrm{Cs}}_{3}{\mathrm{Bi}}_{2}I_{9}{\mathrm{Ti}}_{3}C_{2}\;(e^{-})$$

(3) $$O_{2}+e^{-}\;\to \;•{O_{2}}^{-}$$

(4) $$h^{+}+H_{2}O\;\to \;•\mathrm{OH}$$

(5) $$\mathrm{RhB}\;+\;•{O_{2}}^{-}/•\mathrm{OH}\;\to \mathrm{mineralization}\;\mathrm{products}$$

When the Cs_3_Bi_2_I_9_/Ti_3_C_2_ photocatalyst is exposed to visible light, Cs_3_Bi_2_I_9_ absorbs light and generates electrons in CB and holes in VB. Due to the existence of Ti_3_C_2_, the electrons can be effectively transferred from Cs_3_Bi_2_I_9_ to Ti_3_C_2_, facilitating the separation of e-h pairs in Cs_3_Bi_2_I_9_ and decreasing their direct recombination probability. The electrons in Ti_3_C_2_ combine with O_2_ to generate •O_2_^−^, and the resultant holes in Cs_3_Bi_2_I_9_ are turned into •OH with the aid of water. These •O_2_^−^ and •OH radicals can oxidize organic pollutants into mineralization products.

Generally, the photogenerated holes in the photocatalyst must have enough potential to oxidize water into •OH radicals. This process requires that the hole potential is greater than 1.99 eV. In addition, when the CB potential is less than the redox site of •O_2_^−^ (−0.35 V to +0.94 V) [57,58], then the electrons in CB can react with oxygen to generate •O_2_^−^. In Figure 6b, the VBM of Cs_3_Bi_2_I_9_ is 2.01 eV and the Fermi energy level of Ti_3_C_2_ is −0.13 eV, which both are enough for the generation of oxidation radicals. Based on the scavenger experiment results (Figure 4d), it can be seen that eliminating •O_2_^−^ is more influential on the photocatalysis, specifically that the •O_2_^−^ radicals originated from the transferred electrons on Ti_3_C_2_ play the most important role in the whole degradation process. Without Ti_3_C_2_, a large amount of photogenerated electrons in Cs_3_Bi_2_I_9_ cannot be transferred and have to directly recombine with holes and take a path of radiative emission, after which the oxidative radicals reduce and the photocatalytic efficiency correspondingly decreases.

## 4. Experimental

### 4.1. Materials

Cesium acetate [Cs(OAc), 99.9%], octadecene (ODE, 90%), iodotrimethylsilane (TMS-I, 97%), titanium aluminum carbide (Ti_3_AlC_2_, 98%), and MG were purchased from Roan. Bismuth acetate [Bi(OAc)_3_, 99.99%] was purchased from Bidepharm. Oleic acid (OA, AR) was purchased from Aladdin. Oleylamine (OLA, 70%) and BQ (97%) were purchased from Macklin. Hexane (C_6_H_14_, 97%, AR), hydrofluoric acid (HF, 40%, AR), IPA (99.7%, AR), sodium sulfate anhydrous (Na_2_SO_4_, 99%, AR), ethanol anhydrous (C_2_H_5_OH, 99.8%, GR), acetone (CH_3_COCH_3_, 99.5%, AR), EDTA-2Na (99%, AR), and MB were purchased from Xilong Scientific. RhB was purchased from Tianjin Guangfu fine chemical research institute. Nafion solution (5%) was purchased from Chengxin Science and Technology. The purified water came from Wahaha. All reagents and solvents were used as received without any purification.

### 4.2. Synthesis of Ti_3_C_2_

First, 2 g of Ti_3_AlC_2_ was slowly added to 15 mL HF and 5 mL deionized water with constant stirring to etch the Al in Ti_3_AlC_2_ completely. Then, the mixed solution was stirred at room temperature for 5 h. The stirred solution was centrifuged to collect the precipitate, and the precipitate was washed with deionized water to a pH value of about 6. Finally, the Ti_3_C_2_ powder was obtained after being dried at 60 °C for 24 h in an oven.

### 4.3. Synthesis of Cs_3_Bi_2_I_9_ and Cs_3_Bi_2_I_9_/Ti_3_C_2_

**Figure 7:**
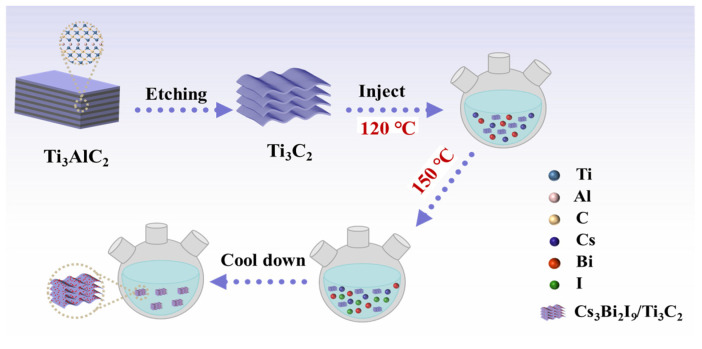
Schematic diagram of the synthesis process of Cs_3_Bi_2_I_9_/Ti_3_C_2_ composite.

A total of 0.14 mmol Cs(OAc), 0.2 mmol Bi(OAc)_3_, 1.5 mL OA, and 0.37 mL OLA were dissolved in 6 mL ODE. It was heated to 120 °C under an N_2_ atmosphere. After the temperature was maintained for 30 min, the solution temperature was raised to 150 °C and 0.2 mL TMS-I was rapidly injected to react for 1 min, before being cooled in an ice water bath for 30 s. The reacted solution was centrifuged at 8000 rpm for 15 min to obtain the precipitate. The precipitate was dispersed in hexane and centrifuged at 5000 rpm for another 15 min, and finally the precipitate was collected and dried in an oven at 60 °C for 24 h to obtain Cs_3_Bi_2_I_9_ powder. The synthesis process of Cs_3_Bi_2_I_9_/Ti_3_C_2_ is similar and shown in Figure 7, only differing in the fact that 3 mg Ti_3_C_2_ was added into the Cs(OAc)/Bi(OAc)_3_ solution when the temperature was heated to 120 °C.

### 4.4. Characterization

An X-ray diffractometer (XRD, Miniflex 600, Rigaku, Tokyo, Japan) was used to study the crystal structure of Cs_3_Bi_2_I_9_, Ti_3_C_2_, and composite material Cs_3_Bi_2_I_9_/Ti_3_C_2_. Its scanning angle range was 5−60°, and the scanning speed was 2°/min. The elemental compositions of Ti_3_C_2_, Cs_3_Bi_2_I_9_, and Cs_3_Bi_2_I_9_/Ti_3_C_2_ were characterized using an American X-ray photoelectron spectrometer (XPS, Thermo Scientific K-Alpha, Waltham, MA, USA). The morphology of Ti_3_C_2_ was analyzed using a Czech scanning electron microscopy (SEM, TESCAN MIRA LMS, Brno, Czech Republic). The microstructures of Cs_3_Bi_2_I_9_ and Cs_3_Bi_2_I_9_/Ti_3_C_2_ were characterized and the lattice spacing was determined using a Japanese high-resolution transmission electron microscope (TEM, JEM-2100F, Hitachi, Tokyo, Japan). The valence electron structure of Cs_3_Bi_2_I_9_ was measured using UV photoelectron spectroscopy (UPS, ESCALAB 250Xi, Thermo Scientific, Waltham, MA, USA). The UV-vis absorption spectra of photocatalysts and organic pollutants were measured using a UV spectrophotometer (UV2700, Shimadzu, Kyoto, Japan), and the measurement wavelength range was 200~800 nm. The photoluminescence (PL) spectrum of the sample was measured using a fluorescence spectrometer (Edinburgh FL/FS900 Carry Eclipse) from Agilent Technologies, Santa Clara, CA, USA. An excitation of 380 nm was used, and the collected emission rage was 400–800 nm. The contact angle between Cs_3_Bi_2_I_9_, Cs_3_Bi_2_I_9_/Ti_3_C_2_, and water was measured using an SDC-200 angle measuring instrument of Donguan Shengding Precision Instrument, Dongguan, China.

### 4.5. Photocatalytic Activity Tests

The photocatalytic performance of the photocatalyst was investigated by using a xenon lamp (PLS-SXE300, 300 W, Perfectlight, Beijing, China) equipped with a filter (λ > 420 nm) as the visible light source and RhB (20 mg/L), MB (10 mg/L), and MG (20 mg/L) as pollutants. As usual, 50 mg of the photocatalyst and 50 mL of dye solution were mixed in a 250 mL beaker and stirred in the dark for 30 min to achieve an adsorption–desorption equilibrium between the contaminant and photocatalyst. Then, the solution was placed under the xenon lamp 4 cm away, and every 5 min, 3 mL solution was taken out to determine the current concentration of the organic dye. The pollutant concentration was determined by the UV-vis spectrophotometer. Cyclic photocatalysis experiments were carried out by repeatedly collecting photocatalysts via centrifugation and drying. In addition, free radical trapper experiments were carried out using IPA, EDTA-2Na, and BQ as scavengers of •OH radicals, •O_2_^−^ radicals, and the holes (h^+^), and then the photocatalytic effects were observed to determine which groups participated in the photocatalytic process.

### 4.6. Electrochemical Tests

Transient photocurrents and electrochemical impedances were measured using a standard three-electrode electrochemical workstation (CHI 660E, Shanghai, China). The electrolyte was Na_2_SO_4_ solution (0.2 M) and the light source was the same xenon lamp. The sample, graphite, and AgCl electrodes were used as the working, comparison, and reference electrode, respectively. The photocatalyst was deposited on the fluorine-doped tin oxide (FTO) electrode. A clean FTO electrode was obtained by sequential soaking and sonication in acetone, washing in purified water, and sonication in anhydrous ethanol. The mixture of 10 mg sample, 100 μL purified water, 100 μL anhydrous ethanol, and 30 μL nafion was sonicated for 20 min and then it was added dropwise with a pipette gun to form an orange-red coating on the electrically charged side of the FTO electrode. After drying in an oven, the sample-coated FTO electrode was then used for tests.

## 5. Conclusions

In this work, we successfully synthesized a Cs_3_Bi_2_I_9_/Ti_3_C_2_ photocatalyst, and the incorporation of Ti_3_C_2_ led to the effective separation of electrons and holes in Cs_3_Bi_2_I_9_ and thus greatly improved the photocatalytic efficiency of Cs_3_Bi_2_I_9_. Under visible light, Cs_3_Bi_2_I_9_/Ti_3_C_2_ can photodegrade 97.3% RhB, 96.0% MB, and 98.8% MG in 30 min, almost 1.6 times faster than sole Cs_3_Bi_2_I_9_. This work shows that Ti_3_C_2_ is an effective electron seceder when working with Cs_3_Bi_2_I_9_ and also reveals the great potential of the photocatalytic application of non-lead perovskite.

## Figures and Tables

**Table 1:** Comparison of photodegradation ability of perovskite photocatalysts.

Photocatalysts	Contaminants	Light Source	Efficiency	Ref.
CsPbI_3_/g-C_3_N_4_	5 mg/L RhB in water	Visible	99% in 80 min	[48]
CsPbI_3_/BiMoO_6_	5 mg/L RhB in water	Visible	95.8% in 90 min	[49]
Au-ZIF-67-CsSnBr_3_	10 mg/L MG in water	UV	60% in 210 min	[50]
CsPbBr_3_/UiO-66	10 mg/L MO in water	Visible	90.5% in 90 min	[51]
Cs_3_Bi_2_I_9_/g-C_3_N_4_	20 mg/L 4-NP in water	Visible	100% in 180 min	[52]
Cs_3_Bi_2_I_9_/g-C_3_N_4_	10 mg/L TC in water	Visible	92% in 120 min	[52]
Cs_3_Bi_2_I_9_/g-C_3_N_4_	10 mg/L MB in water	Visible	98.7% in 150 min	[52]
Cs_3_Bi_2_I_9_/g-C_3_N_4_	10 mg/L MO in water	Visible	85.1% in 150 min	[52]
Cs_3_Bi_2_Br_9_	20 mg/L RhB in water	UV	100% in 100 min	[53]
Cs_3_Bi_2_Br_9_	20 mg/L MB in water	UV	99.3% in 100 min	[53]
Cs_3_Bi_2_I_9_	20 mg/L RhB in water	Visible	93% in 180 min	[14]
Cs_3_Bi_2_Cl_9_	20 mg/L RhB in water	Visible	100% in 100 min	[54]
Cs_3_Bi_2_Cl_9_	20 mg/L MG in water	Visible	100% in 60 min	[54]
Rb_3_Bi_2_Cl_9_	20 mg/L RhB in water	Visible	95% in 100 min	[54]
Cs_2_AgInCl_6_	10 mg/L RhB in ethanol	UV	90.8% in 140 min	[55]
Cs_2_AgInCl_6_	10 mg/L MR in ethanol	UV	92.9% in 70 min	[55]
Cs_3_Bi_2_I_9_/Ti_3_C_2_	20 mg/L RhB in water	Visible	97.3% in 30 min	our
Cs_3_Bi_2_I_9_/Ti_3_C_2_	10 mg/L MB in water	Visible	96.0% in 30 min	our
Cs_3_Bi_2_I_9_/Ti_3_C_2_	20 mg/L MG in water	Visible	98.8% in 30 min	our

## Data Availability

The data can be made available upon reasonable request.
